# THE ASSOCIATION OF MILD TBI WITH EARLY ONSET DEMENTIA IN POST-9/11 VETERANS

**DOI:** 10.1093/geroni/igz038.2435

**Published:** 2019-11-08

**Authors:** Mary Jo Pugh, Hari Krishna Raju Sagiraju, Blessen Eapen, Alicia A Swan, Margaret Wells, Jason R Soble, Janice C Marceaux

**Affiliations:** 1 University of Utah, Salt Lake City, Utah, United States; 2 VA Greater Los Angeles Health Care System, Los Angeles, California, United States; 3 University of Texas San Antonio, San Antonio, Texas, United States; 4 South Texas Veteran Health Care System, San Antonio, Texas, United States; 5 University of Illinois College of Medicine, Chicago, Illinois, United States

## Abstract

Evidence produced by studies using ICD-9 codes to identify dementia suggests that mild traumatic brain injury (mTBI) can accelerate age-related neurodegeneration and dementia risk. However, ICD-9 codes are unreliable in identifying early onset dementia (<65 years; EOD) in civilian (positive predictive value [PPV]=58%) and VA (PPV=28%). From 1,724 Veterans <65years of age with 2 or more dementia diagnoses based on ICD-9 codes recommended by VA Dementia Steering Committee, we validated dementia diagnoses in 153 randomly selected cases using medical chart abstractions and reviews by a neuropsychologist panel. We matched valid cases based on age, sex, race/ethnicity, year of entry to VA care, and branch of service to 2490 controls with no indicators of dementia or cognitive impairment. TBI severity was defined using multiple DoD and VA data sources. We also identified diagnoses for mental (e.g., depression, post-traumatic stress disorder, substance use disorders) and other medical conditions (e.g., stroke) associated with dementia. We used conditional logistic regression to examine the association of TBI severity with EOD controlling for comorbidity. After controlling for mental health and other comorbid conditions mild TBI (mTBI) was significantly associated with validated EOD [aOR( 95%CI): mTBI-4.5(2.4-8.9), moderate/severe TBI-21.3(8.4-54.3)]. Stroke, depression, PTSD, and headache were also associated with higher odds of EOD. These findings suggest that Veterans with mTBI are at risk for dementia, and clinicians should consider brief screening for cognitive dysfunction to ensure that they receive timely treatment to mitigate and address the impact of dementia on the individual, caregiver, family, and health care system

